# Occluder Device Removal and Total Septation of “Swiss-Cheese” Ventricular Septal Defects

**DOI:** 10.1016/j.atssr.2024.05.010

**Published:** 2024-06-04

**Authors:** Andrew K. Morse, Julija Dobrila, Damien J. LaPar, Jorge D. Salazar

**Affiliations:** 1 Children’s Heart Institute, Children’s Memorial Hermann Hospital, University of Texas Health Science Center at Houston McGovern Medical School, Houston, TX, USA

## Abstract

"Swiss-cheese" ventricular septal defects present complex treatment challenges. Despite difficult defect visualization and closure, complete septation is the treatment of choice. We present the case of a 2-year-old with residual apical "Swiss-cheese" ventricular septal defects after failed percutaneous device closure with 2 occluder devices. Surgical removal of 1 device and primary closure of the defects via right apical ventriculotomy resulted in successful complete septation.

Multiple (>4) muscular, or "Swiss-cheese," ventricular septal defects (VSDs) is a rare and serious condition.^1^ Historically, pulmonary artery (PA) banding was the preferred treatment, but its high morbidity and septal hypertrophy have made surgical septation the treatment of choice.^2^ The septation of “Swiss-cheese” VSDs is technically challenging due to the difficulty of visualizing every defect, and several different techniques have been developed including primary closure via right, left, or bilateral ventriculotomies; septal obliteration via right atriotomy; and the double-patch sandwich technique.^3^ Despite advancements, the septation of "Swiss-cheese" VSDs still carries significant risk of mortality and complications such as ventricular dysfunction, residual shunts, and heart block.^4^

Studies on percutaneous occluder devices as a minimally invasive option for “Swiss-cheese” VSDs have highlighted concerns about unmasking VSDs and residual shunts. Current data show that occluder devices are associated with a higher risk of residual shunting compared with surgery when closing single VSDs, and rates of mortality after percutaneous closure of “Swiss-cheese” VSDs have been reported between 14% and 24%, with rates of device failure between 20% and 40%.^1^^,^^5^ Herein, we report the septation of residual “Swiss-cheese” VSDs in a 2-year-old patient after failed closure with percutaneous occluder devices via an apical right ventriculotomy.

A 2.5-year-old male child with residual “Swiss-cheese” muscular VSDs was referred to our institution with heart failure and failure to thrive. At an outside institution, he had undergone PA banding and patent ductus arteriosus ligation via thoracotomy at 3 months of age, device closure of muscular VSDs with a 6-mm Amplatzer Occluder and a 6-mm Amplatzer Duct Occluder II (Abbott Laboratories) at 21 months, and PA de-banding and DeVega annuloplasty of the tricuspid valve (TV) at 23 months.

Preoperative echocardiogram showed moderate-severe left atrial dilatation, mildly dilated mitral valve (MV), and a mildly hypoplastic TV without insufficiency. The devices were in the apical region of the interventricular septum (Figure 1A, Video). There were VSDs 3-4 mm in size in the infundibular and mid-muscular regions, as well as numerous smaller VSDs in the apical region surrounding the devices. Cardiac catheterization revealed a ratio of pulmonary blood flow to systemic blood flow of 2.4:1 and the patient’s PA pressures were half systemic at a mean of 22 mm Hg. The decision was made to pursue complete septation via a right apical ventriculotomy.

**Figure 1:**
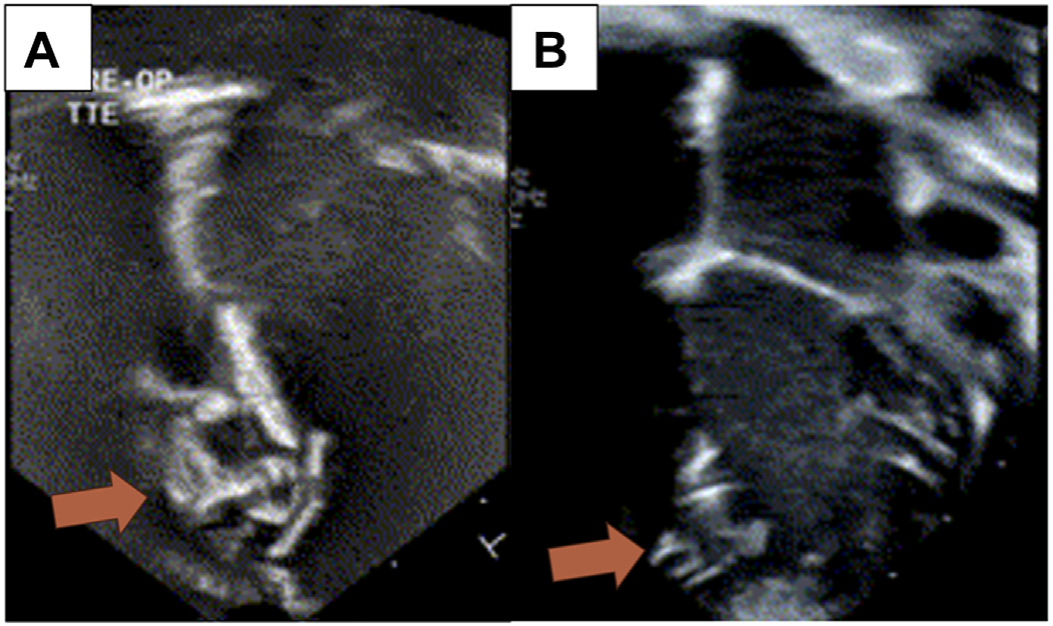
(A) Preoperative and (B) postoperative transthoracic echocardiogram of the apical septal region showing devices and septal defect repair (arrows).

In the operating room, a median sternotomy was performed and the patient was placed on cardiopulmonary bypass at 30°C. Via right atriotomy, the DeVega annuloplasty was taken down before the annular ridge of scar was resected to increase the TV annulus. Via right apical ventriculotomy, the anterior device was removed and the posterior device was left in place (Figure 2). The muscular VSDs surrounding the devices were closed primarily from the left side via the septal opening that remained after device removal. The right ventricle (RV) outflow tract was incised to visualize the infundibular VSD before its closure. The septal opening was obliterated with the anterior free wall of the RV using pledgetted sutures and the posterior device as an anchor (Figure 3). Posterior annular reduction was performed on the MV to ensure e competency.

**Figure 2:**
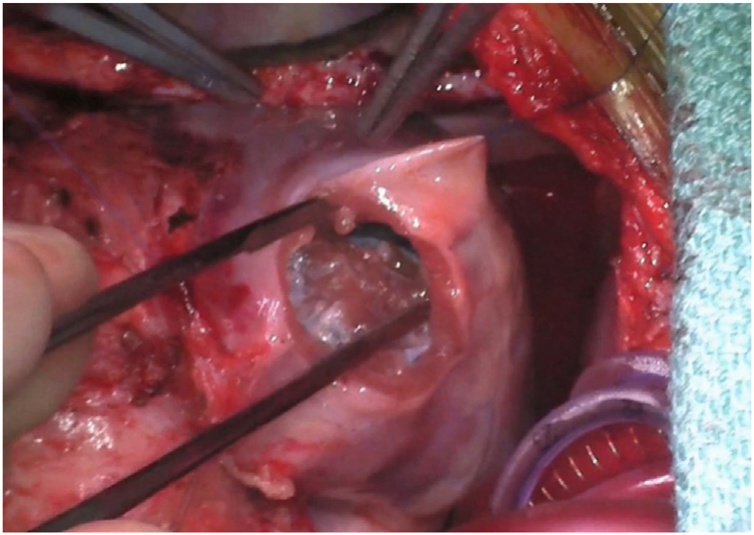
Intraoperative view of the apical septal defect left behind after removal of the anterior occluder device. Ventricular septal defects were visualized and closed from the left ventricular side via the apical defect.

**Figure 3:**
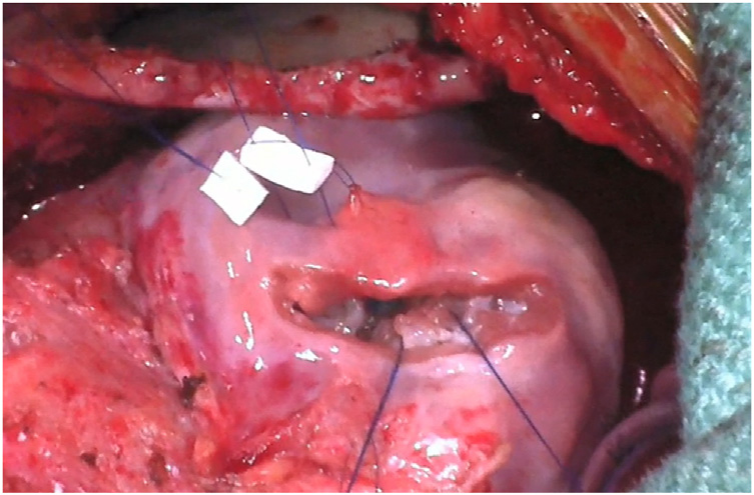
Intraoperative view during obliteration of the apical septal defect with the anterior right ventricular free wall using pledgetted sutures.

Postoperative echocardiogram showed small residual anterior apical muscular VSDs with bidirectional flow that were isolated from the RV cavity (Figure 1B), mild TV and MV regurgitation with no stenosis, mildly depressed left ventricle (LV) function, and normal RV function. Permanent atrial pacemaker leads were placed, his chest was closed, and he was extubated on postoperative day 1. The patient was hemodynamically stable, requiring no vasopressor support, and his hospital course was uneventful before discharge on postoperative day 5. Follow-up echocardiogram on postoperative day 11 showed trivial MV and mild-moderate TV regurgitation with no stenosis, normal RV systolic function, and low normal LV systolic function with septal dyskinesis. An electrocardiogram at that time exhibited normal sinus rhythm with nonspecific intraventricular conduction delays and T-wave changes. At last follow-up, the patient is doing well and continues to enjoy normal daily activities.

## Comment

The surgical repair of apical muscular "Swiss-cheese" VSDs is complex. Traditional primary repair via right atriotomy is challenged by the difficult visualization and closure of individual defects as they are obscured by trabeculations when viewed from the RV aspect. Attempts to divide trabeculations for better visualization often fail and increase the risk of residual shunting.^6^ Percutaneous device occlusion of "Swiss-cheese" VSDs, while less invasive than surgery, has a high failure rate and any subsequent surgical repair after device failure is complicated by the device’s presence.

Recently, ventriculotomy at the LV or RV apex, depending on the defect's anatomy, has emerged as a promising approach for “Swiss-cheese” VSDs. This method allows for the exclusion of all exit points of the VSDs, making it increasingly recognized as a straightforward and effective technique. Long-term data indicating a low risk of ventricular dysfunction following ventriculotomy have also led to more widespread adoption.^7^

We achieved complete septation of apical “Swiss-cheese” VSDs after failed device closure using an apical right ventriculotomy. While the presence of the device initially hindered VSD visualization and closure, the defect left behind after its removal allowed for simpler visualization and closure of the VSDs from the LV aspect due to the finer trabecular muscle structure. Thus, we capitalized on the advantage of visualization from the LV without the need for left ventriculotomy. An additional RV outflow tract incision was required to visualize additional VSDs before closure. To close the large apical defect left behind by the device after the closure of the VSDs, pledgetted sutures were used to sandwich the defect to the anterior RV free wall, similar to a technique previously described by Kitagawa and colleagues.^8^ The device that was not removed was used as an anchor for the pledgetted sutures during this process to reinforce the closure.

In conclusion, the complete septation of apical muscular “Swiss-cheese” VSDs after failed transcatheter occluder device closure can be done safely and successfully via a right apical ventriculotomy with removal of the occluder device and closure of the VSDs from the left side. Given the relatively high failure rate of device closure and preferable long-term outcomes of full surgical repair, complete septation should be pursued in similar cases.
